# ACE2 in the Gut: The Center of the 2019-nCoV Infected Pathology

**DOI:** 10.3389/fmolb.2021.708336

**Published:** 2021-09-21

**Authors:** Yuexin Guo, Boya Wang, Han Gao, Lei Gao, Rongxuan Hua, Jing-Dong Xu

**Affiliations:** ^1^Department of Oral Medicine, School of Basic Medical Sciences, Capital Medical University, Beijing, China; ^2^ Undergraduate Student of 2018 Eight Program of Clinical Medicine, Peking University Health Science Center, Beijing, China; ^3^Department of Physiology and Pathophysiology, School of Basic Medical Sciences, Capital Medical University, Beijing, China; ^4^ Department of Bioinformatics, School of Biomedical Engineering, Capital Medical University, Beijing, China; ^5^Department of Clinical Medicine, School of Basic Medical Sciences, Capital Medical University, Beijing, China

**Keywords:** ACE2, 2019-nCoV, gut, function, biological actions

## Abstract

The 2019-nCoV is a rapidly contagious pneumonia caused by the recently discovered coronavirus. Although generally the most noticeable symptoms are concentrated in the lungs, the disorders in the gastrointestinal tract are of great importance in the diagnosis of 2019-nCoV. The angiotensin-converting enzyme 2 (ACE2), an important regulator of many physiological functions, including blood pressure and nutrients absorption, is recently identified as a vital entry for 2019-nCoV to enter host cells. In this review, we summarize its functions both physiologically and pathologically. We also elaborate its conflicting roles from the clews of contemporary researches, which may provide significant indications for pharmacological investigations and clinical uses.

## Introduction

### General Biological Characteristics of SARS-CoV-2

Sharing many similarities with severe acute respiratory syndrome coronavirus (SARS-CoV) and Middle East respiratory syndrome coronavirus (MERS-CoV), severe acute respiratory syndrome coronavirus-2 (SARS-CoV-2) is also an important member of coronavirus with a single positive-stranded RNA genome (Perlman and Netland, 2009). The entry of it into the host cells requires the membrane fusion between a viral envelope and cell membrane, which is mediated by a viral envelope protein, trimeric spike (S) glycoprotein (Zhu et al., 2019). On engaging a host cell receptor, the receptor-binding domain (RBD) of S1 undergoes hinge-like conformational movements and the determinants of receptor binding are exposed (Wrapp et al., 2020). Further analysis between the entry process of SARS-CoV and SARS-CoV-2 showed the similarity between the hACE2/SARS-CoV-2-C-terminal domain (CTD) and hACE2/SARS-RBD, while the affinity of the former one is fourfold stronger than that of the latter one (Wang et al., 2020).

In the meantime, despite the wide investigation toward the SARS-CoV-2 pathology, no consistent conclusion has been reached (Sridhar and Nicholls, 2021). On the one hand, multiple organ dysbiosis are involved in the COVID-19, which is caused by SARS-CoV-2 and (Wang et al., 2020) increased the difficulty in confirming the exact origin and the transmission pathway. On the other hand, the discrepancy between clinical pathological manifestations and autopsy outcomes makes it even harder to illustrate the pathogenesis and distribution of this nightmare of humanity. One of the theories receiving much approval indicates the endothelial dysfunction to be the prelude of the syndrome. However, although the postmortem analysis of the transplanted kidney by electron microscopy revealed viral inclusion structures in endothelial cells, this process could not be repeated in cell lines in other experiments (Hou et al., 2020; Varga et al., 2020). Studies have corroborated that upon infection in human C2BBe1 intestinal cells expressing a brush border, SARS-CoV-2 can stay persistently and induce the production of interferon (IFN)-α, IFN-β, IFN-λ1, IFN-λ2, and IFN-λ3 more effectively *ex vivo* than that in the lungs (Lee et al., 2020), and the replication of SARS-CoV-2 in the colorectal adenocarcinoma Caco-2 cell line occurs without the cytopathic effect (CPE), contrary to that in human airway epithelial cells (Wurtz et al., 2021). In human intestinal enterocyte cells, the infection seems to be more complicated, often without serious tissue damage, but results in the disruption of cell connections including tight junctions *via* the interaction of the viral E protein and proteins associated with Lin seven-1 (PALS1) (De Maio et al., 2020). Similarly, the viral E protein can also form the homopentameric ion channel affecting the ion transportation, resulting in the elevation of Ca^2+^ concentration in the cytoplasm and serious cell functional and structural impairments (Cao et al., 2020). SARS-CoV-2 is also equipped with another ion-channel protein, Orf3a, which facilitates the export of K^+^ ion from inside the cell and could lead to cellular inflammatory state owing to the leakage (Ren et al., 2020). Furthermore, SARS-CoV-2 could also disturb the RAS system and result in inflammation in various organs, which will be explained in detail in *Interactions via the RAS System* (The main points of this review are summarized and shown in Box 1).

BOX 1 **Key points.**

ACE2 is widely distributed in various organs and tissues, concomitant with the multiple symptoms of 2019-nCoV all over the body. This lays the anatomical basis for the correlation between ACE2 and the virus entry into host cells and suggests the pivotal role it plays in the pathology of both the primary infection and subsequent complications.

ACE2 is known to play important roles in the RAS system, which regulates the blood pressure and associates with some renal and cardiovascular diseases. The RAS system also correlates with gastrointestinal manifestations and results in the alteration of GI microbial components.

Apart from the RAS system, ACE2 also affects the gastrointestinal system by regulating the antimicrobial peptide secretion *via* the mTOR pathway activated by tryptophan and nicotinamide, which is transported *via* ACE2-co-expressed B^0^AT1.

sACE2 and others factors, such as immune cells and cytokines, may account for the spread of SARS-CoV-2 infection. Gut microbes could also regulate the diseases in other organs *via* direct transmission of pathogens or indirectly through its metabolites.

### Epidemiology of SARS-CoV-2

To date, SARS-CoV-2, also known as 2019-nCoV, has affected more than 38,000,000 people worldwide and caused over 1,000,000 deaths (Tu et al., 2020). Common symptoms include fever, cough, and other respiratory disorders, but gastrointestinal symptoms are also witnessed, with the morbidity ranging from 1/10 to approximately 1/3 (Du et al., 2020; Parasa et al., 2020; Velev et al., 2020; Xu et al., 2020). The difference in sample sizes may account for the disease manifestation rate, and other factors, such as age, could influence the statistics, as children and pregnant women always show merely mild manifestations (Ellington et al., 2020; Whittaker et al., 2020; Zhang et al., 2020). Sometimes, the vague and subjective definition of diarrhea initially given by the World Health Organization (WHO) may also be the source of this misunderstanding and disagreement. Besides the alteration in disease morbidity, some gastrointestinal symptoms could also serve as indicative markers in the infection process, as diarrhea and abdominal discomfort set up even earlier than those respiratory disorders and indicate a longer disease duration and worse therapeutic outcome (D'Amico et al., 2020). Some GI symptoms are related to more severe diseases in other organs, coinciding with the previously reported existence of the axis between the gut and other organs (Huang et al., 2020; Zhang et al., 2020; Zhang et al., 2020). In other clinical trials, some patients show positive viral RNA in the feces although the test results in the respiratory system are negative, indicating the possibility of fecal–oral transmission (Xiao et al., 2020). This sets the alarm for the prevention of hospital-acquired infection and arises the controversy considering whether diarrhea could be a certain index for infected detection (Liang et al., 2020).

### Overall Expression of ACE2

Tremendous studies have corroborated the vital roles that the angiotensin-converting enzyme 2 (ACE2) plays a role in the universal entry of 2019-nCoV into the host cells, showing that even a seemingly small amount of virus could result in a relatively large amount of infection (Lamers et al., 2020). Although this dependence is common in many animals, the entry of the virus into mice seems to be an exception, increasing the difficulty in laboratory investigations (Zhou et al., 2020).

Encoded by the 40 kb ACE2 gene located on chromosome Xp22, ACE2 is a close homolog of ACE, which is an important regulator in the renin-angiotensin system (RAS), but has quite the opposite functions of it (Donoghue et al., 2000; Gheblawi et al., 2020). According to the comprehensive analysis of transcriptome datasets in four public databases, including the Tissue Atlas of Human Protein Atlas (HPA), Genotype-Tissue Expression (GTEx), Functional Annotation of Mammalian Genomes 5 (FANTOM5), and Cap Analysis of Gene Expression (CAGE), colon is found to be one of the organs with highest expression of ACE2 mRNA. Antibody-based protein profiles analyzed by HPA show high abundance of the ACE2 protein in the small intestine while relatively lower in the colon (Wang et al., 2020). Studies using immunoprecipitations further convinced that the expression of ACE2 mainly converged on intestinal brush border membranes, providing anatomical basis for the virus entry into the gastrointestinal system (Verdecchia et al., 2020; Zhang et al., 2020). Further investigations confirmed the impact of many factors on its expression level, such as age, inflammatory state, different parts of the intestine, and cell type. This is coinciding with the altered disease manifestations in different subject groups (Bangma et al., 2020), and the lack of the ACE2 expression in the upper GI tract may account for reasons why GI symptoms are not as typical as those in the respiratory system, although the epithelium in the upper GI tract also provides crucial defense against virus infection.

### Two Forms of ACE2 and its Receptor

Discovered in 2000, ACE2 is recognized as a homolog of ACE conserving two domains: the amino-terminal catalytic domain and the carboxy-terminal domain (Tipnis et al., 2000). However, one active site of its catalytic domain—the zinc metallo-peptidase domain—shows 58.2% sequences different from ACE, making it able to convert Ang I to Ang-(1–9) and Ang II to Ang-(1–7), compared with ACE which would generate Ang I to the potent vasoconstrictor Ang II (Corvol et al., 1995).

ACE2 is initially presented on cell membranes, and then after cleaved by a disintegrin and metalloproteinase 17 (ADAM17), it could be shed into blood as soluble ACE2, promoting the conversion of Ang I to Ang-(1–9) and Ang II to Ang-(1–7) (Turner et al., 2002). This cleaving process could be stimulated by Ang II *via* its type 1 receptor (AT1 receptor) although only a relatively small proportion of membrane ACE2 is cleaved in this way. It is believed that circulatory ACE2 is mainly capable of regulating blood pressure as well as maintaining the electrolyte and liquid homeostasis, while membrane ACE2 primarily takes control of multiple organ functions (Perlot and Penninger, 2013).

### Entry and Pathology of COVID-19 Associated With ACE2

As a brief description of the general cell pathology upon SARS-CoV-2 infection has been given in *Epidemiology of SARS-CoV-2*; here, we put our emphasis on its interaction with ACE2 in the gut. In fact, the main infection process has been verified in several studies utilizing different cells, including those from both the lungs and the GI tract (Cleary et al., 2020; Lamers et al., 2020). Single-cell RNA-sequencing (scRNA-seq) analysis showed a strong co-expression of ACE2 and type II transmembrane serine protease (TMPRSS2) in ileum absorptive enterocytes after virus infection, suggesting a pivotal role that ACE2 plays in the virus entry process (Ou et al., 2020). Further investigations using flow cytometry demonstrated the direct bind of ACE2 and SARS-CoV-2 S protein after incubation (Bröer, 2008; Ou et al., 2020). Based on the knowledge that SARS-CoV could employ TMPRSS2 for S protein priming, Hoffmann et al. investigated whether a similar process was used for SARS-CoV-2 (Iwata-Yoshikawa et al., 2019). They noticed that the use of E-64d, a protease inhibitor, also termed loxistatin, inhibited the virus entry process in a dose-dependent manner, while the directed expression of TMPRSS2 rescued the infection (Hoffmann et al., 2020).

To understand the virus reproduction inside infected cells, researchers performed scRNA-seq analysis and found the upregulated expression of ACE2 and interferon-γ receptor 2 (IFNGR2) genes in human type II pneumocytes and macrophages (Mφ) in the lungs. Further investigations focused on the relative alteration of the mRNA level verified the previous suggestion that ACE2 is in fact a kind of interferon-stimulating gene (ISG). Although conclusions above are based on experiments both *in vivo* and *in vitro* in the nasal epithelium, scRNA-seq showed strong correlation between ACE2 and ISGs using the sample metadata from The Cancer Genome Atlas (TCGA) (Bao et al., 2020; Ziegler et al., 2020). However, studies using human small intestinal organoids (hSIOs) found that IFNs (interferons)-Ⅰ and Ⅲ were induced only in a relatively small amount, while an increased level of interferon-stimulating genes (ISGs) was demonstrated by gene oncology analysis (Lamers et al., 2020). Known as antivirus genes, the activation of IFNs initiates a series of protein expressions defending against the virus in almost every stage of infection. One of this is ACE2, and its regulation may account for the decrease in the expression level of ACE2 after the virus entry (Siyu Bai, 2018). The decrease in the ACE2 levels could further lead to an enhancement of the serum angiotensin II (Ang II) concentration, causing multiple organ disorders, including the heart, the lungs, and the kidney (Kuba et al., 2005).

The alterations in the ACE2 level exert a direct influence on the renin-angiotensin system (RAS) spreading among various organs in the body. The transmission of the virus could also initiate a series of cytokine storm in the immune system, being detrimental for varies organs and tissues (Yongzhi, 2021).

In this review, after summarizing the chemical and biological characteristics of ACE2, we analyzed its functions in gastrointestinal diseases and the SARS-CoV-2 infection process. We also summarized its interactions with other organs and the underlying mechanisms. More detailed and targeted investigations would be of great help in understanding the infectious pathology and thus facilitate the clinical treatment.

## ACE2: Regulating the Gastrointestinal Inflammation

Although the most recognizable characteristic of ACE2 is its protection against hypertension, its impact on the gastrointestinal system also deserves attention, as the progress of hypertension is always concomitant with the alterations of gut microbes (Li et al., 2017). Until now, there are generally two mechanisms accounting for this, the first one through the activation of mTOR while the second one *via* Mas.

### Affecting the Tryptophan Absorption (*via* mTOR)

B^0^AT1 (SLC6A19) is a membrane-bound tryptophan transporter widely expressed in the kidney and the intestine (Bröer et al., 2004). Its mutation or loss could result in the occurrence of the Hartnup disorder (OMIM 234500), an autosomal recessive disorder occurring at a frequency of about 1:30,000 in the European population (Bröer, 2009). Under normal conditions, ACE2 is reported to co-express with several amino acid transporters in the microvilli of human small intestine enterocytes, such as B^0^AT1 for tryptophan (Perlot and Penninger, 2013) and signaling threshold-regulating transmembrane adaptor 1 (SIT1) for proline, sarcosine, or betaine (Bröer, 2008; Vuille-dit-Bille et al., 2015). Therefore, it is plausible that the lack of ACE2 could result in the decrease in l-tryptophan absorption and other related diseases. However, some concurring intestinal bowel disease (IBD) symptoms are also witnessed, and some intestinal disorders are also found in the Hartnup disease using radiological methods (Navab and Asatoor, 1970; Camargo et al., 2020). All these findings indicate the potential role of ACE2 in mediating the gut inflammation process. In an attempt to understanding this, (Hashimoto et al., 2012) utilized ACE2 knockout mice and tested the levels of relatively key molecules. According to them, the level of Ang II remains almost unchanged, excluding the role of the RAS system. Then, to demonstrate the role of amino acids, they fed the mice with a Trp + diet and found that the symptoms were markedly relieved. Then, to further confirm the role of tryptophan in mediating gut inflammation, they tested the level of antimicrobial peptides and found that it significantly downregulated in mice fed with a protein-free diet (PFD), rectifying their hypothesis that ACE2 could alleviate the gastrointestinal inflammation by securing the uptake of tryptophan. Moreover, they also demonstrated the role of mTOR activation in the initiation of the antimicrobial peptide release.

Apart from their contribution in showing mTOR activation in the ACE2-Trp antimicrobial peptide pathway, other mechanisms and molecules may also account for this regulation, which is remaining for further elucidation.

### Interactions *via* the RAS System

Discovered in 2000, ACE2 first came to be known as a homolog of ACE, hydrolyzing angiotensin I (Ang I) to generate angiotensin 1–9 and counterbalancing the classic Ang I/ACE2/Ang II pathway in the RAS system (Donoghue et al., 2000). Later, further investigations found that apelin peptides could also be catalyzed by ACE2 (Vickers et al., 2002). Meanwhile, the functions of RAS have extended from those within the kidney and the cardiovascular system in the beginning to the well-distributed ones in many other organs (Hashimoto et al., 2012; Choi et al., 2020; Samavati and Uhal, 2020). These functional researches, in turn, promote the discovery of various downstream pathways mediated by Ang I and Ang II through an angiotensin type 1 receptor (AT1R) and an angiotensin type 2 receptor (AT2R) as well as a Mas receptor (MasR) (Jackson et al., 1988).

It has been demonstrated that besides the functions of regulating systemic blood pressure, these molecules could regulate the physiological condition within the local tissue, such as cell proliferation (Oliveira et al., 2020). The ACE/Ang II/AT1 axis and ACE/AngII/AT2 axis mainly impact the transportation of water and electrolytes, while the ACE2/Ang-(1–7)/MasR axis regulates the nutrient uptake to a large extent (Oliveira et al., 2020). Ang was found to influence the contraction of the intestinal smooth muscle after stimulating ganglia cells in pigs (Page, 1962). Then, using rats’ proximal colon connected with a digital voltmeter, researchers verified the increase in the hydraulic conductivity after injected with angiotensin (De Los Rios et al., 1980). Similar studies also corroborated the regulation of alkaline secretion in the duodenal mucosa using chloralose-anesthetized rats (Ewert et al., 2006), and the nutrient uptake could also be influenced by Ang, as reported by Wong et al. (2007) using immunocytochemistry, Western blotting, and RT-PCR. Besides those functions mediated by AT1R and AT2R, several important pathways have been found to be mediated by the Mas receptor, including the Toll-like receptor 4 (TLR4) controlling the secretion of antimicrobial activities and the PI3K-AKT pathway for cell proliferation. Reduced proliferation of the colonic myofibroblast and ACE2 could be induced by Ang (1–7) in a dose-dependent manner, and the same effect is seen in the secretion of collagen by the colonic myofibroblast (Garg et al., 2020).

Besides, they also play important roles in pathological conditions such as inflammation, oxidation, and fibrosis (Oliveira et al., 2020). Radioimmunoassay showed higher levels of Ang I and Ang II in IBD patients, indicating the protection role of ACE2 in gastrointestinal inflammation (Jaszewski et al., 1990; Garg et al., 2012). Concomitant with this, the use of ACE inhibitors as well as AT1R blockers could suppress the release of pro-inflammatory cytokines and ameliorate colitis in mice (Inokuchi et al., 2005; Santiago et al., 2008).

The apelin peptides, consisting of 77 amino acids, are endogenous ligands of the G protein-coupled receptor, apelin receptor (APJ), and present in vascular endothelial cells, augmenting the cardiac output (Bai et al., 2014). They can be hydrolyzed and cleaved by ACE2 into apelin-13 and apelin-36 (Saeedi Saravi and Beer, 2020). Studies have shown that similar to the normal RAS system, the apelin/APJ system plays important roles in GIT, such as gastric acid secretion, regulation of the appetite and food intake, cell proliferation, cholecystokinin secretion and histamine release, gut–brain axis, and GI motility (Huang et al., 2019). Although most researches did not include this peptide in consideration of the connection between the RAS and gastrointestinal tract (GIT), this is also a potential pathway of great importance and deserves further investigations (Garg et al., 2012).

Apart from the downstream pathways mentioned above that could serve as mediators between the RAS and the immune system, molecules regulating various immune functions are also found to be able to control the ACE2 cleavage *via* its convertase ADAM17 (Wong et al., 2016; Neurath, 2020). By coculturing human aortic endothelial cells (ECs) with human monocytes, Papapetropoulos et al. (1996) reported that monocytes could decrease the ACE activity via the release of cytokines such as TNF-α and IL-1. These findings unveiled the potential relationship between the RAS and the immune system, while more specific interactions within the GIT are still required for clinical diagnosis and treatment.

The gastroenterologists also investigated the impact of drugs that the IBD patients use on their risk of infection. Tissue injury resulted from cytokine storm accounts for much of the symptoms in 2019-nCoV and IBD; some also have indications for comorbidities such as diarrhea (Zhang et al., 2020). Thus, the immunosuppressive drugs used for treating IBD may also be beneficial. As mentioned above, many drugs targeting AT1R, AT2R, and ACE have been found to relieve the symptoms to some degrees, such as CGP42112A and losartan (De Godoy and Rattan, 2006; Ewert et al., 2006). However, considering the wide roles of RAS in almost the whole body, its impact on other systems and organs remains to be seen.

### Direct Binding to Integrins

Integrins are heterodimeric transmembrane receptors that bind to an extracellular matrix, a cell surface, or soluble ligands and send signals to and from the cell cytoplasm. They regulate the cell adhesion, proliferation, differentiation, migration, and apoptosis and play important roles in the development of varies diseases (Hynes, 2002). Studies have found that ACE2 could bind with integrin β1 and block its interaction with microvascular cell adhesion molecule MAdCAM-1 or VCAM-1, alleviating the inflammation process (Danese, 2011). Further analysis corroborated that the lowering proportion of phosphorylated Akt accounts for this inflammation alleviation (Clarke et al., 2012).

### Concerning the Susceptibility of IBDs

**FIGURE 1:**
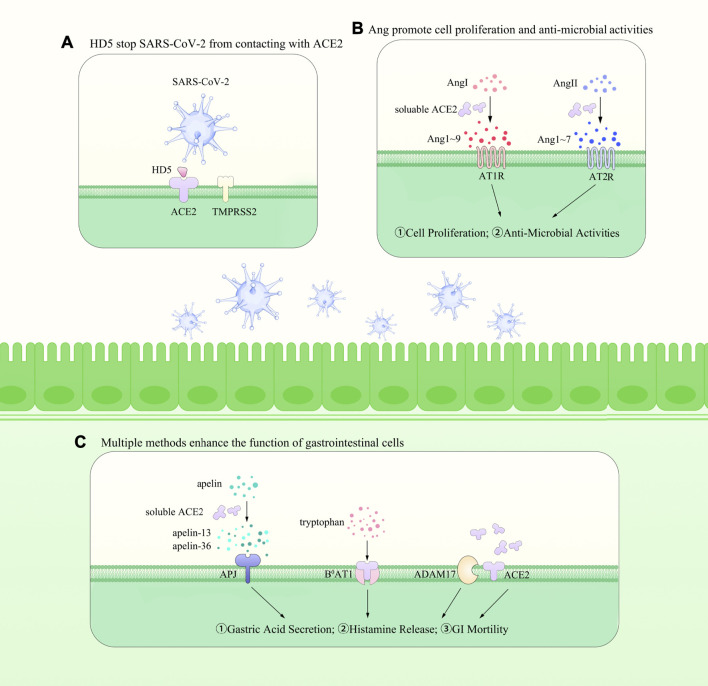
The roles of ACE2 within the gut. In the meantime, it promotes the SARS-CoV-2 entry into the epithelial cells with the association of TMPRSS2 [shown in **(A)**]. Besides, as an important regulator of RAS, it cleaves Ang I to Ang-(1–9) and Ang II to Ang-(1–7) and helps with the maintenance of blood pressure [shown in **(B)**]. ACE2 is widely expressed on the membrane of gastrointestinal epithelial cells and could be cleaved into soluble ACE2 *via* ADAM17. In the meantime, it mediates the transportation of amino acids and is responsible for the apelin peptides [shown in **(C)**]. However, it also promotes the SARS-CoV-2 entry into the host cells, increasing the difficulty in understanding its exact effects within the GIT and in the whole body.

Concomitant with the increased level of ACE2 witnessed at both mRNA and protein levels in IBD patients, studies also demonstrated a higher level of ACE2 in patients with Crohn’s disease (CD) than those with ulcerative colitis (UC) (Ning et al., 2019), suggesting that ACE2 could serve as a protector against intestinal inflammation. However, considering its pivotal role in facilitating the virus entry into host cells, it comes to be a premier question regarding the generally exact role of ACE2 in IBD patients. So far, no clinical evidence shows a higher possibility for IBD patients to be infected, probably due to the relatively low amount of sACE2 compared with those bound on the membrane (Taxonera et al., 2020). However, many auxiliary measures have been taken in prevention of IBD patients against SARS-CoV-2 infection (Bai et al., 2020), beneficial for the prevention of SARS-CoV-2 epidemiology in IBD patients, but it could be an obstacle in investigating the exact impact of ACE2 on the virus entry into IBD patients. As we have showed that sACE2 could help with the entry of the virus while barely helpful for the expression of the key amino acid transporter, it is reasonable to say that membrane bound ACE2 mainly plays a protective role in the prevention of SARS-CoV-2 infection (Taxonera et al., 2020). In the meantime, several drugs targeting ACE2 have been in the process of trials, while human recombinant ACE2 (rhACE2) is also responsible for decreasing the number of the viruses that enter by competing with mACE2 (Annweiler et al., 2020; McKee et al., 2020). Detailed findings concerning the altteration of ACE2 level are summarized in Table 1 and multiple roles of ACE2 have been depicted in Figure 1(as shown above).

**TABLE 1:** Comparison of ACE2 levels among different tissues in typical diseases.

Index	Methods	Tissue	Consequence	Reference
ACE2 level	Immunohistochemical analyses	Terminal ileum and colon	Higher in IBD patients	Vickers et al. (2002)
Average expression of soluble ACE2	Quenched fluorescent substrate–based assay	Soluble level	Higher in IBD patients	Neurath, (2020)
ACE2 activity	Fluorescence-based assay	Inflamed mucosa	Decrease non-inflamed colon in IBD	Vickers et al. (2002)
ACE2:ACE ratio	Quenched fluorescent substrate––based assay	In plasma	Higher in patients with IBD	Neurath, (2020)
ACE2 in inflamed intestine	Tandem mass tag–based shotgun proteomics	Inflamed intestinal areas	Higher in patients (CD)	Bai et al. (2014)

## Indicative Relations With Other Organs

Many GI symptoms shown in 2019-nCoV are also witnessed in many diseases originated in other organs, indicating the existence of potential interactions between them. Besides the axis dominated by bacteria and their metabolites we have discussed above, other potential approaches *via* ACE2 have also been put forward and deserve careful consideration in clinical treatment, which could provide novel targets for disease treatment and tissue repair. In the first place, the loss of smell, a common indicator of neurological disorders, is associated with multiple GI malfunctions (Shen et al., 2010). Some researchers speculate that neurological innervation may account for the anatomical basis, while others reported that the olfactory bulb lesions (OBLs) could rely on intestinal immunodeficiency caused by olfaction loss–induced denervation injury of Peyer’s patches (Firinci et al., 2019; Bostancıklıoğlu, 2020).

Besides, considering the existence of soluble ACE2, it is possible for other systems to be disturbed with the alteration of the ACE2 level in the infectious area, as studies found that the loss of ACE2 could result in functional deterioration of the heart and progression of cardiac, renal, and vascular pathologies (Kuba et al., 2013). Similarly, using immunofluorescence, researchers also witnessed the disrupted gut barrier integrity as well as alterations in microbial components and displacement in diseases seemly restricted in a typical organ (Duan et al., 2019).

Moreover, other possible underlining pathways, such as nerve stimulation and nutrient metabolism, can also result from the intestinal flora alteration and account for the concomitant pathological manifestations (Oliveira Andrade et al., 2017).

**FIGURE 2:**
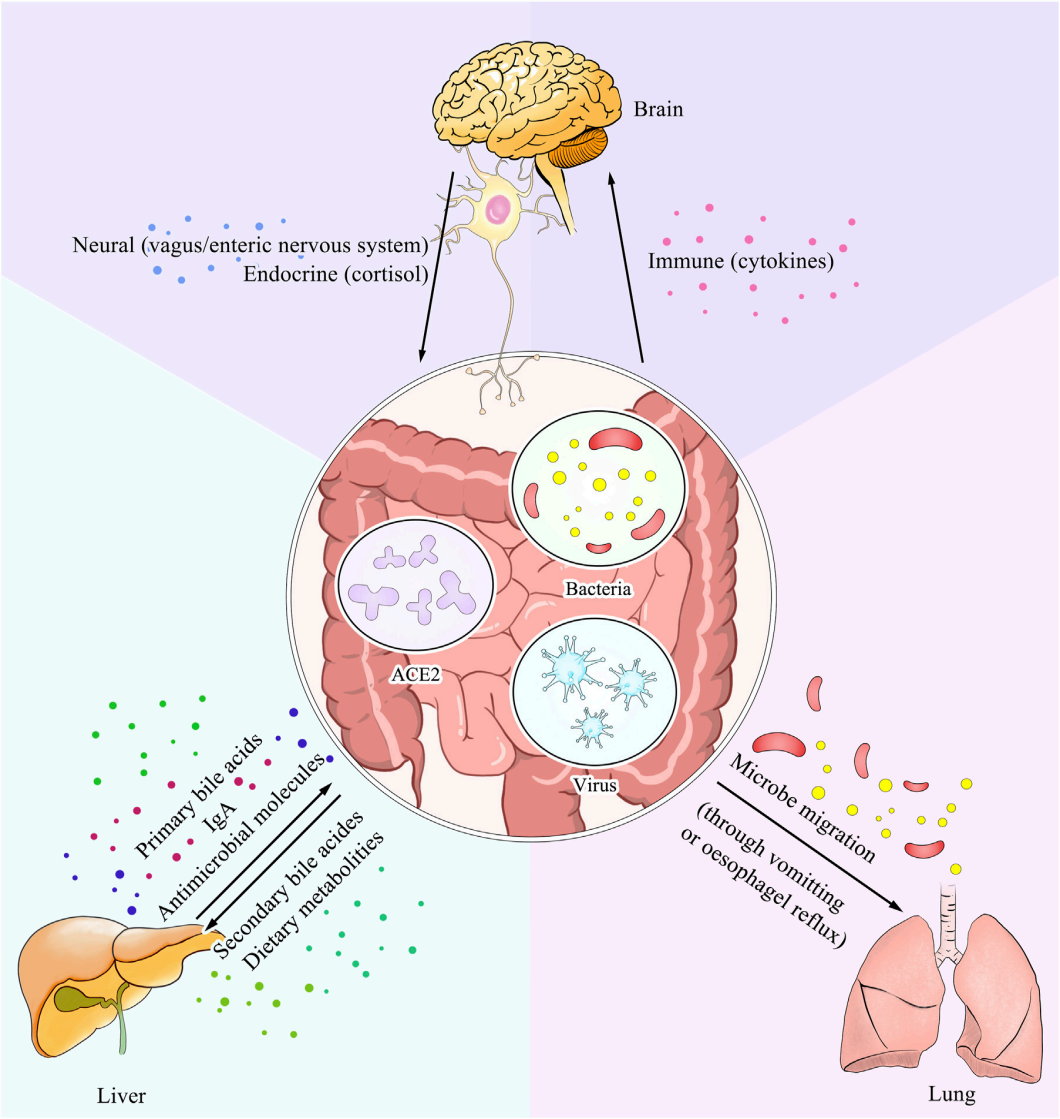
ACE2 serves as a dominant link between the gut and other parts of the body. The expression level of ACE2 within the gut could interfere with the nutrient metabolism and result in alteration of the intestine microbes. Also as a pivotal ligand for the entry of SARS-CoV-2, it has an influence on the virus concentration. Moreover, the soluble form of ACE2 could also transmit to other organs and lead to disturbance of the physical condition. All three ways mentioned above are shown in the picture with explanation of different pathways.

Additionally, the alterations in the number of lymphocytes within one organ may affect that in plasma, which could possibly cause physiological changes in others, as both 2019-nCoV and IBD patients show immune dysregulation characterized by elevated levels of neutrophils and inflammation markers (Boros et al., 2020; Mehta et al., 2020). Similar changes have been observed in other organ diseases, such as *Streptococcus pneumoniae* infection and tubular injury (Adams et al., 2020). Given the ACE2 regulation of the immune system, more detailed research studies with careful variant control are required, especially those performed on typical diseases and cell lines. (Please refer to Boxes 2–4 for detailed information concerning the extrapulmonary symptoms in COVID-19 associated with GI). And Figure 2 provides a vivid description of these interactions.

BOX 2 **Allotriosmia: clinic and epidemiology.**

Allotriosmia is generally used to describe diseases with a total or partial loss of smell. It could be divided into two subsets due to different etiologies: phantosmia referring to the dysfunction in stimulating relative reactions after odors emerge and parosmia for impairment in perceiving the stimulation (Holbrook and Leopold, 2003; Morrissey et al., 2016). Seen in multifactorial quantitative traits controlled by both genetic and multiple diseases, especially concerning impairment to head, allotriosmia is also common in other sensorineural processes, such as upper respiratory infection (URI) (Daramola and Becker, 2015). Epidemiological researches in COVID-19 found that the olfactory dysfunction (OD) is probably the strongest predictor of COVID-19 and for those infected with COVID-19 showed a prevalence of 62%, while OD patients have a positive predictive value of 61% for a positive COVID-19 result (Rocke et al., 2020). Therefore, the complex interactions among OD, COVID-19, and GI tract receive much attention with many investigations being carried out.

BOX 3 **Brief introduction of the etiology and risk factors for extrapulmonary manifestations of 2019-nCoV.**

The etiology of 2019-nCoV includes many elements, and there is an extraordinary complex correlation between each of them. It is really hard to describe them all clearly and deeply with limited words, so we just list several main points about the occurrence of extrapulmonary manifestations of COVID-19. Hematologic tests showed higher levels of inflammatory markers such as C-reactive protein and IL-6, while severe illness was found in those infected who also have concomitant preexisting diabetes and/or obesity (Gupta et al., 2020). Neurologic disorders such as encephalopathy (Poyiadji et al., 2020) and renal dysfunctions including proteinuria (Su et al., 2020) and hematuria are also witnessed (Cheng et al., 2020). Such complications indicate complex interactions among organs in the infection process and call for further researches and experiments.

BOX 4 **COVID-19 and diabetes.**

A large number of observations have shown that COVID-19 patients often experience multi-organ dysfunctions and symptoms are even severer in those with systematic diseases. In a study among 324 hospitalized COVID-19 patients, 55 (16.97%) had diabetes mellitus and showed a significantly higher mortality rate (Acharya et al., 2020). In the meantime, analysis has shown inflammatory signs in the GIT in those patients, concomitant with an increase in the number of harmful metabolites such as lipopolysaccharide. This could result in higher susceptibility to COVID-19, as shown in the epidemiological researches (Terruzzi and Senesi, 2020).

## Host Defense Peptides in Novel COVID-19 Antiviral Therapy

Since the outbreak of COVID-19, researchers have been trying to find effective drugs to block the infection and cure the disease. Among them, antiviral peptides (AVPs), known for their simple primary structure, could serve as the molecular templates and have been evaluated (Mahendran et al., 2020). Among various kinds of AVPs developed in recent years, the HD5 (ATCYCRTGRCATRESLSGVCEISGRLYRLCCR), a natural lectin-like human defensins-5 (HD5) peptide secreted by the Paneth cells in the crypts of Lieberkuhn, could protect the host cells from viral recognition and infection by interacting with glycosylated proteins and lipid components and competitively blocking ACE2 receptors on the host cells (Wang et al., 2020). In the meantime, studies also corroborated the expression of HD5 in other tissues including the human renal proximal tubular epithelial cells, probably accounting for the protective roles evidenced by concomitantly ameliorated clinical symptoms in those areas (Du et al., 2020). Taken together, these findings provide new directions for drug development and bring bright prospect for treatment against COVID-19.

## Conclusion

ACE2 is an enzyme widely distributed in various organs. Apart from the commonly recognized role as an important component in the RAS system, ACE2 also regulates the amino acid uptake and is in control of many downstream immune pathways. Detailed regulating mechanisms of ACE2 are also investigated, as it is demonstrated to be a necessary binding protein for the SARS-CoV-2 entry into host cells. In this review, after giving a brief introduction to epidemiological statistics of COVID-19, we summarized the latest research progress focused on ACE2 and SARS-CoV-2 within the GIT. We also analyzed its role in IBD and relation with diseases in other organs. Based on those analyses, we point out some possible orientations for future investigations and clinical trials. Considering the variety of organs and diseases involved in ACE2-mediated pathological processes, more relevant investigations would be of great benefit for building up a set of effective measurements in the precaution and treatment for 2019-nCoV.
