# Corrigendum: ACE2 in the gut: The center of the 2019-nCoV infected pathology

**DOI:** 10.3389/fmolb.2022.1063507

**Published:** 2022-10-21

**Authors:** Yuexin Guo, Boya Wang, Han Gao, Lei Gao, Rongxuan Hua, Jing-Dong Xu

**Affiliations:** ^1^ Department of Oral Medicine, School of Basic Medical Sciences, Capital Medical University, Beijing, China; ^2^ Undergraduate Student of 2018 Eight Program of Clinical Medicine, Peking University Health Science Center, Beijing, China; ^3^ Department of Physiology and Pathophysiology, School of Basic Medical Sciences, Capital Medical University, Beijing, China; ^4^ Department of Bioinformatics, School of Biomedical Engineering, Capital Medical University, Beijing, China; ^5^ Department of Clinical Medicine, School of Basic Medical Sciences, Capital Medical University, Beijing, China

**Keywords:** ACE2, 2019-nCoV, gut, function, biological actions

In the published article, there were errors in **Affiliations** 1, 3 and 5.

In **Affiliation 1**, instead of “Department of Oral Medicine, Basic Medical College, Capital Medical University, Beijing, China,” it should be “Department of Oral Medicine, School of Basic Medical Sciences, Capital Medical University, Beijing, China.”

In **Affiliation 3**, instead of “Department of Physiology and Pathophysiology, Basic Medical College, Capital Medical University, Beijing, China,” it should be “Department of Physiology and Pathophysiology, School of Basic Medical Sciences, Capital Medical University, Beijing, China.”

In **Affiliation 5**, instead of “Department of Clinical Medicine, Basic Medical College, Capital Medical University, Beijing, China,” it should be “Department of Clinical Medicine, School of Basic Medical Sciences, Capital Medical University, Beijing, China.”

The authors apologize for this error and state that this does not change the scientific conclusions of the article in any way. The original article has been updated.

